# Pronated foot and reactive balance: A preliminary comparative study of older women

**DOI:** 10.1371/journal.pone.0331928

**Published:** 2025-09-16

**Authors:** Chatanun Chinpeerasathian, Akkradate Siriphorn, Praneet Pensri

**Affiliations:** Department of Physical Therapy, Faculty of Allied Health Sciences, Chulalongkorn University, Bangkok, Thailand; Cairo University Faculty of Physical Therapy, EGYPT

## Abstract

**Background:**

Older adults with pronated foot may face greater challenges in maintaining balance, which increases their risk of falling. Reactive balance, which refers to the ability to restore stability following an unforeseen disturbance, is a key component in evaluating fall-related postural control. Evaluating reactive balance can provide insights about balance capabilities and potential fall risks in this population.

**Objective:**

This study compared the reactive balance (center of mass displacement and reaction time) between older women with and without pronated foot.

**Methods:**

Thirty-two older women comprising 16 with bilateral pronated foot and 16 without pronated foot participated in the study. To assess reactive balance, a three-dimensional motion analysis was conducted. Each participant was equipped with 29 retroreflective markers and compensatory stepping corrections were performed in the forward direction. Independent t-test was used to compare the center of mass displacement and reaction time between the two groups.

**Results:**

The older women with pronated foot exhibited significantly slower reaction times than those without pronated foot (p = 0.017). However, no significant difference was determined for the center of mass displacement between the two groups (p = 0.367).

**Conclusion:**

This study indicated that older women with pronated foot had significantly prolonged reaction times, suggesting an impairment in reactive response. However, the lack of significant differences in the center of mass displacement between those with and without pronated foot suggest that although older women with pronated foot maintain balance similar to those without pronated foot, their delayed reaction times may hinder the ability to make quick, involuntary stepping adjustments, potentially increasing fall risks.

## Introduction

Foot and ankle disorders in older adults are linked to reduced mobility, impaired balance, increased disability, and a higher risk of falls and fractures [1–3]. Older adults are likely to have conditions in their ankles and feet including toe deformity (60%), bunion (37.1%), flat feet (19.0%), and overlapping toes (15.6%) [1–4]. During standing, older adults exhibit a significantly lower foot arch compared to younger adults, with older women demonstrating a notably flatter arch than their male counterparts [5]. The feet widen and flatten with age, and the fat padding on the soles of the feet becomes stiffer, dissipates more energy when compressed, and recovers more slowly after the load is removed [3].

Pes planus, also referred to as flatfoot or pronated foot, is a biomechanical condition marked by the flattening of the medial longitudinal arch, excessive subtalar joint pronation, rearfoot eversion, and dorsiflexion coupled with forefoot abduction [6,7]. Several biomechanical factors contribute to pronated foot posture, including laxity of the spring ligament, elongation of the plantar fascia, tightness of the gastrocnemius and peroneal muscles, and weakness of the posterior tibialis muscle [8]. Flat feet can lead to damage of surrounding tissues such as ligaments or tendons and alterations in the movements of the proximal joints in the foot as well as the ankle, knee, and hip) [8,9]. Damaged ligaments or tendons impair proprioceptive perception and contribute to poor postural control [10]. Previous studies have found that pronated feet contribute to loss of balance [10,11]. Furthermore, older adults with pronated feet experience a shift in foot pressure and an increased area for center of gravity [12].

Balance control is required for three types of functional tasks and activities: steady-state, reactive, and proactive balance. Steady-state balance refers to the ability to maintain control over the center of mass (COM) relative to the base of support in stable and unvarying conditions [13]. Proactive, or anticipatory, balance refers to the ability to engage the legs and trunk in preparation for voluntary movements that may compromise stability, allowing for the maintenance of postural control and movement efficiency. Delayed or absent anticipatory muscle activity can lead to loss of balance and falls [13]. Reactive balance control refers to the ability to regain stability following an unexpected external disturbance, enabling the body to restore equilibrium and prevent loss of balance [13]. For example, walking and tripping over an obstacle or being bumped in a crowd necessitate the activation of multiple muscles in the legs and trunk to regain a COM stable position relative to the base of support. A fall can result from the inability to rapidly generate and apply appropriate corrective muscle forces to regain balance. Thus a reactive balance assessment (RBA) is commonly used to evaluate reactive balance control [13].

Older adults undergo biopsychosocial changes that affect their daily living and can lead to various problems, particularly falls. Falling is a common accident among older individuals and is more likely to occur in those with associated factors, such as pronated foot [14]. Individuals with pronated foot experience deficits in balance and impaired joint position sense [15]. However, there have been limited examinations of reactive balance in older adults with pronated foot, specifically regarding their capacity to return to stability after an unexpected perturbation. This study aimed to fill this gap by comparing reactive balance assessments between older women with and without pronated foot.

## Materials and methods

### Research design

The present study was a preliminary comparative study. The researcher screened older women with and without bilateral pronated foot. Demographic data was obtained from all individuals who met the screening inclusion criteria. The Foot Posture Index (FPI) was utilized to assess and categorize the foot posture of all participants included in this study. The FPI is a validated clinical tool that quantifies foot posture along a continuum from highly supinated to highly pronated, using a 6-item observational scoring system. Each criterion is scored on a scale from −2 to +2, resulting in a total possible score ranging from −12 (highly supinated) to +12 (highly pronated). A total score of 0 to +5 indicates normal foot posture [16].

**Fig 1:**
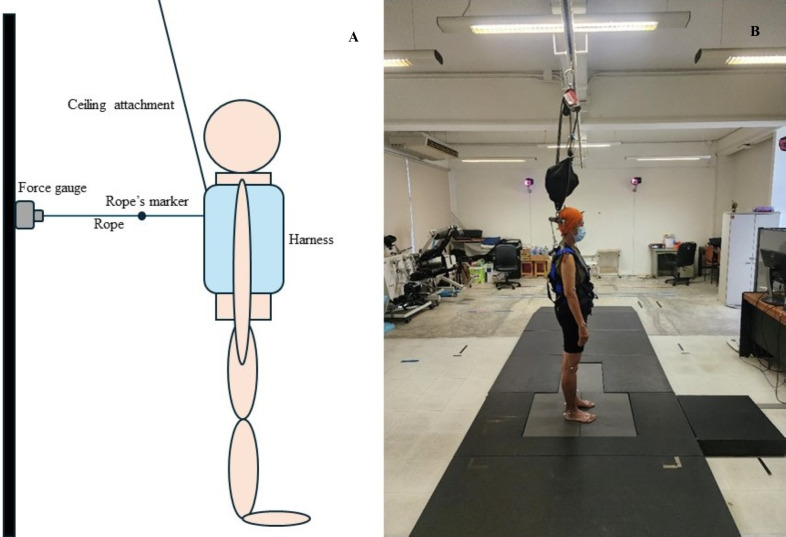
Reactive balance assessment setting. (A) Graphic illustration of the experimental setup. (B) Actual setup in the testing environment.

This study was approved by the Internal Review Board of Chulalongkorn University (COA. 091/66) and complied with all relevant national regulations and institutional policies. All the participants provided written informed consent prior to data collection. The individual depicted in Fig 1 provided written informed consent to publish the image with the manuscript.

### Sample size

The sample size was determined using G*Power software (version 3.1.9.4, Heinrich-Heine-Universität Düsseldorf, Germany) based on an anticipated effect size of 0.50. With a statistical power of 95% and an alpha level of 0.05, the estimated required sample size was 26 participants (13 per group). Considering a 20% dropout rate, the final total sample size was adjusted to 32 participants, with 16 assigned to each group.

### Participants

Data were collected between July 2023 and February 2024. The 32 participants were recruited from the Bangkok metropolitan area through advertisements. All participants were thoroughly informed about the testing procedures before their participation. The participants were divided into two groups: 16 older women with bilateral pronated foot and 16 older women with a normal foot arch (CTRL).

The inclusion criteria were: 1) woman aged between 60 and 70 years (sex as a controlling factor and age considerations for fall testing), 2) able to walk without assistive devices, 3) FPI value ranging from 0 to +5 without foot pain for the CTRL group and +6 to +9 without foot pain for the pronated foot group (Higher scores ≥+10 were excluded to avoid including potentially pathological conditions, which could confound experimental outcomes), 4) able to step forward in the lean and release test, and 5) normal mental status assessed by the Thai Geriatric Depression Scale.

Potential participants were excluded if they had at least one of the following criteria, 1) body mass index > 30 kg/m², 2) leg length discrepancy, 3) history of lower extremity fracture or surgery, 4) a diagnosis of diabetic neuropathy, cancer, neuromuscular disease, or infectious disease, or 5) history of lower extremity joint arthritis and receiving treatment for it.

### Reactive balance assessment

A RBA assesses balance in a dynamic state during unexpected external perturbations in four directions: forward, backward, and sideways right and left without losing balance, stepping, or reaching for assistance [17]. The RBA evaluates postural instability and serves as a screening tool for identifying individuals at an increased risk of falling [13].

The lean and release test was employed for the RBA, which is a widely used method for evaluating reactive balance ability. This clinical assessment evaluates reactive postural responses and compensatory stepping strategies in response to an externally induced loss of balance [17,18]. This study used three-dimensional (3D) motion analysis (Motion Analysis Corp., Santa Rosa, CA, USA) for the RBA. Eight cameras with flash rates of 120 Hz were used. The motion analysis software (Cortex version 8.1) comprises three main functions: calibrating the capture volume, tracking and identifying marker positions within the calibrated 3D space, and processing data for compatibility with other analytical software. The marker trajectories and analog signals were smoothed using a sixth-order low-pass Butterworth filter, with cutoff frequencies set at 5 Hz for marker data and 50 Hz for analog signals, respectively. This study used 30 reflective markers (29 on the participant and one on the rope) [19]. COM displacement and reaction time were calculated from raw motion analysis files using a custom Python script executed in Visual Studio Code (version 1.85, Microsoft Corp., Redmond, WA, USA) [19]. Fig 1 displays the RBA settings used in this study.

COM displacement during the RBA was defined as the average distance traveled between the initiation of weight release and the participant’s return to a stable, upright position on both feet. Similarly, reaction time was defined as the duration between the initial release of body weight and the participant’s successful reestablishment of a stable standing posture. The initiation of weight release was identified when the marker on the rope exhibited movement of at least 0.8 mm after the rope was severed. Stability was considered reestablished when the participant returned to a normal pelvic posture and upright standing position, with both feet securely in contact with the ground, while the movement of the rope marker remained below 0.8 mm for five consecutive frames [19].

Before data collection, test-retest reliability for this RBA setting was conducted. The intraclass correlation coefficient (ICC) of COM displacement was 0.79 and the reaction time was 0.83 [19]. These values demonstrated high reliability (ICC > 0.75) for both outcome measurements.

### Research protocol

The assessment was conducted at the Motor Control and Motion Analysis Research Laboratory, Department of Physical Therapy, Faculty of Allied Health Science, Chulalongkorn University, Bangkok, Thailand. Prior to gathering data, the researcher contacted every participant to provide preparation information, which included eating a meal 2 hours before data collection, not drinking alcohol or coffee for 24 hours before data collection, sleeping at least 6 hours the night before data collection, and avoiding the use of lotion or oil on their skin on the day of data collection. Furthermore, all participants were required to disclose any drugs taken at the time of data collection. The main researcher explained the objectives and study process to all the participants

To assess reactive balance using a 3D analysis, 29 reflective markers were placed on anatomical landmarks of each participant in accordance with the Helen Hayes marker set [20]. The participants had a safety suspension device wrapped around their bodies during the assessment to prevent falls. After being fully placed with the markers and firmly suspended, they were instructed to walk along a 10-meter walkway at a self-selected pace, determined through 2–3 trials to ensure familiarity with the markers. The forward direction (anteroposterior) was used to investigate RBA in this study. A rope was attached between the digital force gauge (SMAT SENSOR, aostorer-1542650011, Shenzhen, China) and the participant’s body via a fixed belt positioned at the posterosuperior half of the dorsum, near the inferior angle of the scapula [21,22]. For the test, the participants maintained a stable bipedal stance on the force platform with their eyes open. They were instructed to stand upright with their arms at their sides and their feet positioned at shoulder-width apart. Subsequently, they gradually leaned forward, and once the load reached 10% of their body weight, as measured by the digital force gauge, it was unexpectedly released. Each participant received the following instructions: “Stand with your feet shoulder-width apart and your arms at your sides. Lean forward against the rope”. When the loading rope was released, they could take a step to avoid falling and return to stability. In cases of balance loss, the participant was supported by a harness attached to the ceiling, in addition to receiving physical assistance. For this protocol, only one trial was conducted to avoid the effects of fatigue anticipatory reactions, and learning.

### Statistical analyses

IBM SPSS Statistics (version 22.0; IBM Corp., Armonk, NY, USA) was used for quantitative data analyses. The participant demographic data of both groups are presented as means and standard deviations for the numerical data. The Shapiro–Wilk test was conducted to assess the normality of data distribution for each variable, and the results indicated that both variables were normally distributed. Consequently, independent *t*-tests were employed for statistical analysis, with a significance level of p < 0.05. Additionally, the observed power and effect size (Cohen’s *d*) were calculated to assess the strength and reliability of the findings. The effect sizes were interpreted as small (Cohen’s *d* = 0.2), medium (Cohen’s *d* = 0.5), and large (Cohen’s *d* = 0.8) [23].

## Results

A summary of the 32 participant demographics confirmed no significant differences between the pronated foot and CTRL groups regarding age, height, weight, or body mass index (Table 1).

**Table 1:** Clinical characteristics of the two study groups.

	Pronated foot (n = 16)	CTRL (n = 16)	p-value
**Age (year)**	65.1 ± 3.7	64.6 ± 2.8	0.633
**Weight (kg)**	59.1 ± 9.3	58.7 ± 9.3	0.900
**Height (cm)**	158.7 ± 4.4	158.4 ± 5.5	0.867
**BMI (kg/m**^**2**^)	23.1 ± 3.2	22.3 ± 2.8	0.848

Data were analyzed using independent t-tests and are expressed as mean ± standard deviation.

*Significance p < 0.05.

**Fig 2:**
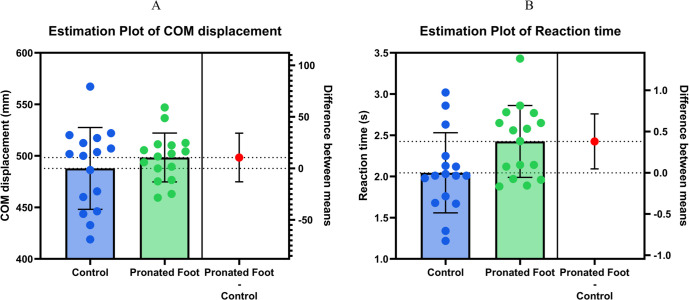
Estimation plots comparing center of mass displacement and reaction time between control and pronated foot groups. Note*: Estimation plots depicting the results of comparing (A) COM displacement and (B) reaction time between the control group and the pronated foot group. Each plot displays individual data points for both groups (blue for control, green for pronated foot), with bars representing group means ± standard deviation. The right panel in each plot shows the mean difference between groups (red dot) with 95% confidence intervals. COM displacement is shown in millimeters, and reaction time in seconds. A statistically significant difference between groups was observed only for reaction time.

Table 2 lists the mean COM displacement measurements and reaction times of the two groups. The raw data corresponding to these variables are provided in the supporting information (S1 Dataset). The pronated foot group had no significant difference in COM displacement compared with the CTRL group (p = 0.367). However, the pronated foot group had a significantly slower reaction time than the CTRL group (p = 0.027). Fig 2 illustrates Estimation plots comparing COM displacement and reaction time between control and pronated foot groups.

**Table 2:** Results of center of mass displacement (mm) and reaction time (s) between the two groups.

Outcomes	Pronated foot (n = 16)	CTRL (n = 16)	t-value	p-value	Mean difference	95% CI	Effect size (Cohen’s d)	Observed power
COM displacement (mm)	498.4 ± 23.7	487.9 ± 39.7	0.92	0.369	10.59 ± 11.57	[-13.03, 34.21]	0.32	0.18
Reaction time (s)	2.4 ± 0.4	2.0 ± 0.5	2.33	0.027*	0.38 ± 0.16	[0.047, 0.714]	0.82	0.62

Data were analyzed using independent t-tests and are expressed as mean ± standard deviation. 95% CI = 95% confident interval.

*Significance p < 0.05.

## Discussion

This study investigated the differences in reactive balance adjustments between older women with and without pronated foot when responding to an unexpected loss of balance. Our findings indicated the pronated foot group demonstrated significantly slower reaction times than the CTRL group. However, there was no significant difference in COM displacement between the two groups. This suggests that although older adults with pronated foot can maintain balance similar to those without pronated foot, their slower reaction times may impair their ability to perform efficient involuntary stepping adjustments, potentially increasing their fall risks.

When considering the outcomes, the pronated foot group had significantly slower reaction times than the CTRL group. The reactive control mechanism primarily relies on two systems, the somatosensory and vestibular systems, which are responsible for assessing the intensity and characteristics of the stimulus and generating appropriately scaled postural responses [24,25]. These postural responses are primarily polysynaptic spinal reflexes and supraspinal responses [24]. The pronated foot group regained stability slower than the CTRL group. This suggests that pronated foot affected the reactive response, particularly reaction time. A systematic review and meta-analysis synthesized findings from multiple studies and concluded that individuals with pronated foot typically exhibit reduced balance and increased postural sway compared to those with normal foot structure [26]. These results align with biomechanical research suggesting that excessive pronation associated with flatfoot impairs the efficiency of force transfer during stance [27]. Additionally, inadequate arch support may contribute to delayed muscle activation in response to perturbations, further impairing postural control and balance [28]. This compromised support can reduce proprioceptive feedback, delaying the body’s ability to initiate corrective responses [29]. These biomechanical factors are consistent with our observation of prolonged reaction times in the pronated foot group.

Several studies have reported that aging affects postural control, and older adults with more physiological deficits have more problems with postural control [30–33]. In addition, a previous study found that older persons have a slower response when returning to stability than younger controls [30]. The older adults had reaction times that were 23% slower than those of younger adults during forward support-surface translations and also showed a reduced capacity to effectively modulate step length and direction under time-critical conditions [30]. Poor reactive control in older adults may result from a combination of factors, including sensory deficits, muscle weakness, cognitive decline, psychological factors such as fear of falling, and neural impairments [30]. The slower reaction times observed in our pronated foot group may be compounded by age-related declines in reflex speed and muscle responsiveness. These age-associated deficits in reactive stepping are clinically relevant, thereby elevating the risk of falls. In the context of our study, which examined the relationship between foot posture and balance performance, it is plausible that slower response times in older adults may be further exacerbated by suboptimal foot biomechanics. This is clinically significant, as pronated foot has been linked to a higher risk of recurrent falls in older adults. These findings underscore the importance of considering both neuromuscular and biomechanical factors in fall prevention strategies for older adults. Interventions that target not only balance training but also foot posture correction may offer additive benefits in improving reactive stepping responses and reducing fall risk.

Nevertheless, this study revealed no significant difference in COM displacement between the pronated foot and CTRL groups. While some studies have reported increased center of pressure displacement in pronated foot individuals during dynamic tasks, our results showed no significant difference in COM displacement between groups, possibly due to compensatory strategies such as increased reliance on hip movements in older adults [34,35]. Reactive balance-correcting responses involve several strategies, including the ankle strategy, which modulates ankle torque to reposition the center of pressure beneath the foot, allowing the COM to return to equilibrium by enabling the body to function as an inverted pendulum, shifting weight through ankle joint movement. The hip strategy involves counter-rotating trunk segments relative to the COM to counteract perturbation-induced changes in angular momentum, facilitating a return to stability. The stepping strategy involves taking a step to increase the base of support, thereby enhancing stability and preventing loss of balance [36]. The lack of significant differences in COM displacement in our study may reflect the unique characteristics of our experimental paradigm, which involved a slip-like perturbation that likely prompted older adults to rely more on hip strategies rather than farther step adjustment. This interpretation is supported by research showing that, as ankle proprioception and function decline with age, older adults increasingly depend on hip proprioceptive information and hip-centered strategies to maintain balance during dynamic and challenging conditions [37,38]. Consequently, the magnitude of COM displacement did not differ significantly between older adults with and without pronated foot, suggesting that a pronated foot structure does not directly impair COM movement in this population.

The reaction time variable exhibited a large effect size (Cohen’s *d* = 0.82), indicating a substantial and clinically meaningful difference between the pronated foot and control groups. This value surpasses the conventional threshold for a large effect as defined by Cohen’s criteria and is further supported by benchmarks commonly used in rehabilitation research [23]. The difference in reaction time was statistically significant (*p* = 0.027), and the observed power was moderate (0.62), suggesting a reasonable probability of detecting a true effect despite the study’s limited sample size. However, the statistical power did not reach the commonly accepted standard of 0.80 [39], underscoring the need for replication with a larger cohort to enhance the robustness of these findings. In contrast, for the COM displacement variable, the effect size was small (Cohen’s *d* = 0.32), and the group difference was not statistically significant (*p* = 0.367). The observed power for this variable was notably low (0.18) [39], indicating insufficient power to detect small effects. Consequently, the non-significant findings for COM displacement should be interpreted with caution. Future research should consider increasing sample size or employing more sensitive assessment tools to better detect potential differences between groups.

Currently, there is no widely published study specifically comparing reaction time or step-balance responses in older adults with pronated foot versus those without pronated foot using reactive or stepping balance assessments. Most research on reactive balance in older adults, including those with a history of falls, has focused on general populations or those with neurological or musculoskeletal impairments. When compared to other work assessing reactive or stepping balance in older adults, our findings align with studies showing that delayed reaction times and altered neuromuscular responses are key risk factors for falls. For example, recent studies using perturbation-based balance training or the stepping threshold test have demonstrated that older adults with a history of falls exhibit significant differences in reactive stepping responses, including delayed muscle activation and altered joint kinematics, compared to non-fallers [30,40,41]. However, these studies have not specifically addressed the impact of pronated foot on reaction time or stepping responses. The available literature on reactive balance in older adults highlights that delayed activation of hip and ankle muscles, as well as slower reaction times, are associated with increased fall risk [30,41]. In this study, older adults with pronated foot demonstrated slower reaction times, mirroring the delayed responses seen in fall-prone populations, even though differences in COM displacement were not observed. This suggests that the biomechanical alterations associated with pronated foot may contribute to a profile of delayed corrective responses similar to that seen in other high-risk older adults.

### Strengths and limitations

This study has several strengths. It was the first to investigate reactive balance responses in older adults with pronated foot and provides novel insights into this population’s biomechanics. The application of motion analysis enabled precise measurement of balance, emphasizing the critical role of biomechanics in maintaining postural stability.

However, this study has a few limitations. First, the study focused exclusively on older women, thus the findings may not apply to older men. Second, the age range of the participants was limited to 60–70 years, which may restrict the applicability of the results to other older populations. Third, this study focused solely on reactive balance responses and did not include assessments of neurophysiological or somatosensory factors, such as proprioceptive acuity or electromyographic activity. This limitation restricts the interpretation of underlying neural coordination and certain components of postural control mechanisms that may contribute to the observed outcomes. Fourth, the reactive balance assessment only used the anteroposterior (forward) direction, leaving the mediolateral and backward balance responses unexamined. Finally, the current study’s sample size limited power for detecting small effects, particularly for COM displacement.

## Conclusions

This study highlights the effect of pronated foot on reactive balance in older women and emphasizes the crucial role of biomechanics in stability. Specifically, older women with pronated foot exhibited significantly prolonged reaction times compared with their counterparts without pronated foot, which suggests a potential impairment in reactive balance responses within this population. This pioneering study establishes a foundation for future research on comprehensive balance assessments and targeted interventions for older adults with pronated foot.

## Supporting information

S1 Dataset Raw data of center of mass displacement and reaction time measurements. (XLSX)
